# Clinical risk factors for recurrence of pelvic organ prolapse after primary native tissue prolapse repair

**DOI:** 10.1007/s00508-021-01861-8

**Published:** 2021-04-30

**Authors:** Barbara Bodner-Adler, Klaus Bodner, Greta Carlin, Oliver Kimberger, Julian Marschalek, Heinz Koelbl, Wolfgang Umek

**Affiliations:** 1 Department of General Gynecology and Gynecologic Oncology, Medical University of Vienna, Währinger Gürtel 18–20, 1090 Vienna, Austria; 2 Department of Anesthesiology, Medical University of Vienna, Vienna, Austria; 3 Department of Endocrinology and Reproductive Medicine, Medical University of Vienna, Vienna, Austria; 4 Department of Special Gynecology and Obstetrics, Karl Landsteiner Institute, Vienna, Austria

**Keywords:** Pelvic organ prolapse, Recurrence, Native tissue repair, Risk factors, Surgical outcome

## Abstract

**Objective:**

To define potential risk factors for recurrence of prolapse.

**Methods:**

This short report included all women who presented with recurrence of prolapse as well as without any recurrence signs after a vaginal approach of native tissue prolapse repair at an urogynecological center in Austria.

**Results:**

A total of 124 recurrence cases and 64 women with no signs of recurrence after their index prolapse surgery were included. Multivariate analysis identified advanced preoperative POP‑Q stage (pelvic organ prolapse-quantification) as an independent risk factor for postoperative recurrence of prolapse (*p* = 0.045).

**Conclusion:**

Initial proper preoperative counseling is of particular importance to modulate patients’ expectations after prolapse surgery.

## Introduction

Pelvic organ prolapse (POP) is defined as “falling or downward displacement of the uterus and/or the different vaginal compartments and their neighboring organs such as bladder, rectum or bowel” [1]. General known risk factors contributing to prolapse are childbirth, collagen abnormalities, increasing age, a chronic increase in intra-abdominal pressure and so on [2]. Treatment options include either pessary placement or surgical repair, whereas around 10% of women undergo prolapse surgery at some time in their lives [3]. The identification of risk factors for POP recurrence appears crucial for the best management of women with this condition. The objective of this study was to identify potential risk factors for prolapse recurrence after index surgery in a cohort of Austrian women.

## Patients and methods

This short report includes 188 documented cases of patients who presented for follow-up after an index prolapse surgery at the department of general gynecology and gynecologic oncology, Medical University of Vienna (MUVI) with recruitment between March 2004 and October 2018. The study was approved by the ethics committee of Medical University of Vienna (EK No. 2186/2019).

Recurrence of prolapse was defined as follows: objective recurrence (prolapse reaching or going below the level of the hymen) or subjective recurrence (symptoms or wish for retreatment).

Planned index surgery included either vaginal hysterectomy with modified uterosacral ligament (USL) suspension as part of standard care for symptomatic POP or sacrospinous hysteropexy or transvaginal anterior/posterior repair (colporrhaphy) in cases of uterus preservation. All index procedures were performed as native tissue repair with the patient under general or spinal anesthesia.

### Statistical analysis

The χ^2^-test was used for the comparison of categorical variables between the two groups and Student’s *t-*test for continuous variables. Additional multivariate logistic regression analysis was conducted to evaluate the association of these clinical variables with postoperative prolapse recurrence. A *p* value < 0.05 was considered statistically significant. The SPSS system (IBM, Armonk, NY, USA, version 25) was used for the calculations.

## Results

### Study group

In this study 124/188 (66%) women with postoperative recurrence signs were compared with 64/188 (34%) postoperative follow-up cases without recurrence signs after initial prolapse surgery. Mean age of all patients was 68 ± 12.9 years (range 34–98 years), mean body mass index (BMI) was 27.8 ± 4.5 kg/m^2^, and mean follow-up duration was 7.55 ± 4.05 years. During this study frame, index surgery consisted of 149 (79%) vaginal hysterectomies (+ modified USL suspension) and 39 (21%) uterus-preserving prolapse surgeries.

### Recurrent cases

Median age at time of recurrence was 70.23 years (range 48–97 years) and median BMI was 28 kg/m^2^ (range 19–42 kg/m^2^). Mean Oxford scale for a voluntary pelvic floor muscle contraction was 1.85 (± 0.751) and 32/124 cases (26%) had stage II prolapse, 70/124 (56%) stage III and 22 (18%) stage IV prolapse.

### Comparison between patients with and without recurrence

Chronic obstructive pulmonary disease (COPD) as well as postmenopausal status were factors that were statistically significantly more common in the recurrence group compared to patients without recurrence (*p* = 0.031; *p* = 0.001). Furthermore, women with recurrence were statistically significantly older and a diminished Oxford scale was observed (*p* = 0.001). The BMI, parity, mode of delivery, uterus preservation, smoking and hypertension did not differ between the two groups (*p* *>* 0.05).

Multiple logistic regression analysis was conducted in order to define independent risk factors for prolapse recurrence. Advanced POP‑Q stage remained an independent risk factor for recurrence of prolapse postoperatively (Table 1).

**Table 1:** Multivariate logistic regression analysis with independent risk factors for recurrence of prolapse

Variable	OR	95% Confidence interval	*p* value
Age	0.970	0.936–1.006	0.098
BMI	0.964	0.896–1.036	0.318
Menopause	0.954	0.886–1.034	0.425
Parity	1.076	0.831–1.393	0.578
POP‑Q	0.621	0.367–1.049	0.045 *

* Statistically significant; *p* < 0.05

*OR* odds ratio, *POP‑Q* pelivc organ prolapse quantification, *BMI* Body mass index

## Summary

In our opinion, the identification of risk factors for postoperative prolapse recurrence appears to be crucial and helps the clinician to divide patients into low-risk and high-risk recurrence cases with potential implications for treatment modalities. In the current study, initial advanced POP‑Q stage was independently associated with prolapse recurrence. This information is important as it could help in clinical practice to counsel patients in an adequate way and to modulate patient’s expectations.
